# An extremely patient-friendly and efficient stimulation protocol for assisted reproductive technology in normal and high responders

**DOI:** 10.1186/s12958-018-0335-0

**Published:** 2018-03-05

**Authors:** Chen-Yu Huang, Guan-Yeu Chen, Miawh-Lirng Shieh, Hsin-Yang Li

**Affiliations:** 1 Division of Reproductive Endocrinology and Infertility, Department of Obstetrics and Gynecology, Taipei Veterans General Hospital, 201, Shih-Pai Road Section 2, Taipei, 112 Taiwan, Republic of China; 2 Division of Obstetrics and Gynecology, Faculty of Medicine, School of Medicine, National Yang-Ming University, Taipei, Taiwan, Republic of China; 3 Institute of Clinical Medicine, National Yang-Ming University, Taipei, Taiwan, Republic of China

**Keywords:** Patient-friendly, Long-acting FSH, PPOS

## Abstract

**Background:**

The use of oral progestin has been shown to effectively prevent luteining hormone (LH) surge during ovarian stimulation with daily human menopausal gonadotropin injections. This study was aimed to investigate the efficacy of long-acting follicle stimulating hormone (long-acting FSH; corifollitropin alfa, Elonva®) use in progestin-primed ovarian stimulation for normal and high responders undergoing IVF/ICSI.

**Methods:**

This is a retrospective and proof-of-concept study. We developed an extremely patient-friendly protocol to be applied to forty-five normal or high responders, in which a single injection of corifollitropin alfa (Elonva®) was administered and medroxyprogesterone acetate (MPA) was taken orally every day from the day after Elonva injection to the day of trigger. Seven days after Elonva injection, folliculometry and hormone tests were performed, followed by short-acting daily FSH/LH injections, if needed, until the day before trigger. Duration of stimulation, number of injections and visits before trigger, incidence of premature LH surge, the number of oocytes retrieved, fertilization rate, cleavage rate, the rate of day 2 good embryos available, and cumulative ongoing pregnancy rate per retrieval were assessed.

**Results:**

The average age of the population was 34.7 years. Duration of stimulation was 9.4 days in average. Before trigger, only 3.6 injection shots and 1.4 visits were needed on average. There was no case of premature LH surge. Number of oocytes retrieved was 13.7, fertilization rate was 79.04%, cleavage rate was 91.11%, and day 2 good embryo rate was 64.34%, in average respectively. There was no case of ovarian hyperstimulation syndrome. The cumulative ongoing pregnancy rate per oocyte retrieval achieved a satisfactory level as 53.1%.

**Conclusions:**

Our protocol consisting of long-acting FSH injection and oral MPA preventing LH surge reduces the number of injections and visits to an extreme and achieves a satisfactory reproductive outcome, and, therefore, is a really patient-friendly and effective approach to ovarian stimulation.

## Background

Prevention of premature luteinizing hormone (LH) surge is the vital component of controlled ovarian stimulation for IVF/ICSI cycles, which is caused by increasing plasma estradiol produced by multiple growing follicles. In the early days, the GnRH agonist down-regulation protocol, which comprises plenty of injections and is therefore named long protocol, was the mainstream to prevent LH elevation [1]. Over the past decade, it was gradually replaced by GnRH antagonist protocol, which is characterized by rapid suppression of LH with fewer injections and reduced risk of ovarian hyperstimulation syndrome (OHSS) [2]. More recently, with improvements in techniques of cryopreservation and thawing, i.e., vitrification of embryos and oocytes, luteal phase stimulation has been adopted [3–5] and achieved satisfactory pregnancy outcomes [6–8]. Stemming from the experiences of luteal phase stimulation, moreover, progestin-primed ovarian stimulation (PPOS) was developed, in which oral progestogen was taken since the first day of ovarian stimulation in the early follicular phase to prevent LH surge; therefore, injections of GnRH analogues can be omitted [9, 10].

Daily injection of FSH/LH was needed in conventional ovarian stimulation. Utilizing recombinant DNA technologies, one single injection of long-acting follicular stimulation hormone (FSH), wherein the FSH β-subunit is extended by a carboxy-terminal peptide (CTP) of the human chorionic gonadotropin (hCG) β-subunit [11], is able to substitute as the first seven daily FSH injections with comparable outcomes in assisted reproduction [12]. Inspired by aforementioned evolutions, we designed an extremely patient-friendly protocol by the combination of long-acting FSH for ovarian stimulation and oral progestin for LH rise prevention. Previous studies on ovarian stimulation protocols using either endogenous or exogenous progestogen to suppress LH surge all utilized daily gonadotropin injection such as human menopausal gonadotropin (HMG) or recombinant FSH (rFSH) [13]. Whether long-acting FSH can be applied in PPOS has not been reported. In this paper, we reported a very patient-friendly and efficient protocol in which an average of 3.6 shots and 1.4 visits were needed before trigger, and this newly developed protocol achieved a cumulative pregnancy rate per retrieval above 50%.

## Methods

### Study setting and patients

A retrospective study was conducted at the Center for Reproductive Medicine in Taipei Veterans General Hospital, and processed by chart review. All patients were counseled and informed consents for the stimulation protocol and related procedures were provided by infertility specialists. Patients with one of the following conditions were not eligible for this protocol: (i) age above forty years, (ii) hypogonadotropic hypogonadism, (iii) basal FSH above 10 IU/L, (iv) antral follicle count (AFC) below 7, (v) body mass index (BMI) above 30, and (vi) uterine abnormalities.

### Treatment protocol of ovarian stimulation and oocyte retrieval

**Fig. 1:**
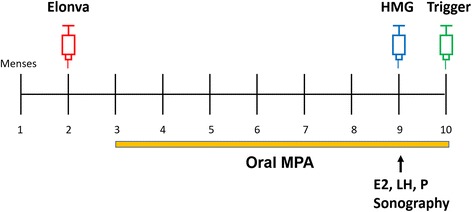
Treatment protocol of ovarian stimulation. On the menstrual cycle day 2, 3 or 4, ovarian stimulation was started with a single injection of corifollitropin alfa (Elonva®; MSD), of which the dosage was determined by the patient’s body weight (150 μg for > 60 kg and 100 μg for ≦ 60 kg). Medroxyprogesterone acetate (MPA) (Provera® 5 mg/tablet; Pfizer) 5 mg twice a day was initiated orally from the day after Elonva injection. Seven days after Elonva injection, follicle development was monitored by transvaginal sonography as well as serum hormone levels of E2, LH and P. The patients received trigger at night if at least three leading follicles reached above 17 mm in diameter, and the final tablet of MPA was taken in the morning of the trigger day. If the folliculogenesis was insufficient for trigger, additional HMG (Menopur®, Ferring) 150 ~ 225 IU/day would be administered for days until the requirement for trigger was met

A simplified schematic description of the treatment protocol is shown in Fig. 1. On the menstrual cycle day 2, 3 or 4, transvaginal sonography and serum hormone levels (FSH, LH, estradiol [E2], progesterone [P]) were checked. If the patients met the aforementioned criteria, ovarian stimulation was started on the same day with a single injection of corifollitropin alfa (Elonva®; MSD), of which the dosage was determined by the patient’s body weight (150 μg for > 60 kg and 100 μg for ≦ 60 kg; [14]). Medroxyprogesterone acetate (MPA) (Provera® 5 mg/tablet; Pfizer) 5 mg twice a day was initiated orally from the day after corifollitropin alfa injection. Seven days after corifollitropin alfa injection, follicle development was monitored by transvaginal sonography as well as serum hormone levels of E2, LH and P. The patients received trigger at night if at least three leading follicles reached above 17 mm in diameter, and the final tablet of MPA was taken in the morning of the trigger day. If the folliculogenesis was insufficient for trigger, additional HMG (Menopur®, Ferring) 150 ~ 225 IU/day would be administered for days depending on the prediction according to the measurement on stimulation day 8. If necessary, follicle monitoring would be performed again every 2–3 days to assess whether the requirement for trigger was met. Triggering was performed by subcutaneous injection of Triptorelin (Decapeptyl® 0.1 mg/ml; Ferring) 0.2 mg with or without hCG 1500~ 6500 IU (Pregnyl®; MSD or Ovidrel®; Merck Sereno), depending on the risk evaluation for early onset OHSS.

Oocytes were retrieved 34–38 h after triggering, followed by in vitro fertilization (IVF) and/or intracytoplasmic sperm injection (ICSI) according to the conditions of the sperm. All embryos were vitrified at pronuclear stage, or on day 2, or on day 3, or on day 5 after retrieval depending on the number of fertilized oocytes available.

### Endometrial preparation for frozen-thawed embryo transfer

In frozen-thawed embryo transfer (FET) cycles, oral estradiol valerate (Estrade® 2 mg/tablet; Synmosa) 6 mg twice per day was prescribed from menstrual cycle day 2~ 4 onwards. After oral Estrade supplement had been taken for 10 ~ 14 days, endometrial thickness was measured via transvaginal ultrasound. In addition, hormone measurement (E2, LH, P) was done to confirm that no spontaneous follicle growth and ovulation occurred. Once the endometrial thickness was greater than 7 mm, oral Estrade supplement was continued and the patient began to receive vaginal micronized progesterone gel 90 mg twice a day (Crinone® 8% 90 mg/applicator; Merck Serono) and vaginal micronized progesterone soft capsules 400 mg per night (Utrogestan®100 mg/capsule; Besins) until either ten weeks of gestation or confirmed failure of pregnancy. The timing of thawing and transfer of cryopreserved embryos was determined according to the stage they were vitrified, synchronized with the duration of progesterone exposure to the endometrium. Embryos frozen at the pronuclear stage were warmed after one day of vaginal progesterone administration, followed by transfer one or two days later based on the number of viable embryos. Embryos vitrified on day 2 or day 3 or day 5 post oocyte retrieval were thawed after 2 days or 3 days or 5 days of luteal support, respectively, and were transferred on the same day. Serum β-hCG level was tested after 14 days of luteal support. If the serum β-hCG titer was above 10 IU/L, transvaginal ultrasound was performed three weeks later to detect the site of gestational sacs along with the fetal heart beat.

### Outcome measures

Demographic variables recorded for each patient included the following items: age, BMI, serum levels of basal E2, P, FSH and LH, AFC, and the indication for IVF/ICSI treatments. Parameters in terms of ovarian stimulation, oocytes, embryos, and pregnancy outcomes from FET were listed below: duration of stimulation, the number of shots and visits before trigger, incidence of premature LH surge, the number of oocytes retrieved, fertilization rate, cleavage rate, the rate of good embryos available, incidence of OHSS, and cumulative ongoing pregnancy rate per retrieval. In the study, peak E_2_ levels and LH levels on the trigger day were not necessarily measured due to freeze-all strategy.

A serum LH concentration exceeding 10 IU/L or rising above twice the basal level before trigger day was considered as premature LH surge. Embryos developing to 2–4 cells on one day after pronuclear stage with a grade of I or II morphology [15], were considered as good embryos. Cumulative ongoing pregnancy rate was counted as pregnancies ≧10 weeks with intrauterine fetal heart beat detected divided by the number of retrieval cycles whose embryos were all transferred or confirmation of intrauterine pregnancies with active fetal heartbeat.

## Results

From February to June in 2017, we have applied long-acting FSH in PPOS with MPA followed by embryo vitrification in 45 normal or high responders. The average age was 34.7 years (range, 28–39 years). The average BMI was 21.4 kg/m^2^ (range, 17.51–27.31 kg/m^2^). The basal AFC was 19.5 in average (range, 8–64). The basal FSH was 6.43 IU/L (range, 4.1–9.2 IU/L) and the basal LH was 4.296 IU/L (range, 0.57–11.22 IU/L), in average. The average basal E2 was 33.19 pg/ml (range, 16.8–61.7 pg/ml). All the demographic data were presented in Table 1. Indications for IVF/ICSI were also shown in Table 1.

**Table 1:** Demographic data of patients (*n* = 45)

	Mean ± standard deviation
Age (years)	34.7 ± 2.4
BMI (kg/m^2^)	21.4 ± 2.51
AFC	19.5 ± 11.7
Basal E2 (pg/ml)	33.19 ± 10.71
Basal FSH (IU/L)	6.43 ± 1.23
Basal LH (IU/L)	4.296 ± 2.090
Indications for IVF/ICSI	
Endometriosis	6
Male factor	18
PCOS	8
Tubal factor	5
Unexplained	5
Combined factors	3

Duration of stimulation was 9.4 days on average (range, 8–15). The average number of injections before trigger was 3.6 shots (range, 2–9). The average number of visits between Elonva shot and trigger was 1.4 (range, 1–2). There were no premature LH surge for all cases. The mean number of retrieved oocytes was 13.7, and the mean fertilization rate was 79.04% (Table 2). The mean cleavage rate and good embryo rate were 91.11% and 64.34%, respectively. There was no case of OHSS.

**Table 2:** Stimulation and oocyte/embryo outcomes

	Mean ± standard deviation
Duration of stimulation (days)	9.4 ± 1.4
No. of injections before trigger (shots)	3.6 ± 1.3
No. of visits between Elonva shot and trigger	1.4 ± 0.5
Premature LH surge	0
No. of oocytes retrieved	13.7 ± 7.8
Fertilization rate (%)	79.04 ± 17.61
Cleavage rate (%)	91.11 ± 10.54
Day 2 good embryo rate (%)	64.34 ± 21.43
OHSS	0

Till submission of this work, 32 patients have either transferred all their cryopreserved embryos without pregnancy or achieved intrauterine pregnancy with good fetal heartbeat. Seventeen of these 32 patients conceived through FET and continued to be pregnant at 10 weeks of gestation with good fetal heartbeat. Therefore, the cumulative pregnancy rate per ovum pickup was 53.1%.

## Discussion

Previous studies confirmed that progestogen administration during follicular phase slowed LH pulse frequency, augmented pulse amplitude, and reduced mean plasma LH levels as compared with those in untreated women studied at the same cycle phase [16]. In animal models, the suppression effect of progestogen on pre-ovulatory GnRH/LH surge is mediated by progesterone receptors in the hypothalamus, and the inhibition is reversible after progestogen discontinuation [17, 18]. Progestogen-induced suppression of LH surge has been applied to develop oral contraceptives, but has not been utilized in ovarian stimulation for IVF until significant improvement of cryopreservation/thawing results brought about by vitrification. “Freeze all strategy” is the prerequisite of PPOS. More and more benefits were discovered about FET under superior techniques of vitrification, including reduced risk of OHSS without compromising implantation rate [19].

MPA has been validated as an effective oral substitute for GnRH analogue injections to prevent LH peak. The use of GnRH antagonist may not completely block the positive feedback of E2 during ovarian stimulation for normal responders [20], and the incidence of untimely LH surge in GnRH antagonist protocols was reported between 0.34% [21] and 35% [22] in different practices. As for the first study presenting MPA use to block LH elevation, a low premature LH surge rate (1/150) was observed in the MPA group. The only case of premature luteinization indicated that in the presence of high basal E2 levels (85 pg/ml), MPA could not prevent imminent LH surge on day 5 of stimulation; however, subsequent folliculogenesis was not affected and no further LH surge occurred (4 oocytes were retrieved from 5 follicles and four embryos were vitrified) [9]. In our study, the basal E2 ranged from 16.8 to 61.7 pg/mL, and we arbitrarily started MPA from one day after corifollitropin alfa injection. As a result, no premature LH surge occurred. In further investigations by the aforementioned group in China, there was zero case of premature LH surge in PCOS patients receiving daily MPA 10 mg for PPOS [23], as well as in normal responders co-administered with either 4 mg or 10 mg MPA per day during ovarian stimulation [24]. Poor ovarian reserve has been noted as a predominant risk factor for a breakthrough LH surge in antagonist protocol [21]. As regards patients with diminished ovarian reserve in PPOS, the incidence of spontaneous LH surge under MPA 10 mg per day was 1.0% in the study including 204 poor responders [25].

Corifollitropin alfa is an rFSH analogue composed of the human FSH α-subunit and a hybrid subunit consisting of the carboxyl-terminal peptide of the β-subunit of hCG coupled with the FSH β-subunit, which is able to sustain follicle-stimulating activity equal to the circulating FSH level above the threshold necessary for multiple follicle growth throughout an entire week [26]. Seven daily FSH injections can thus be replaced by one single shot of corifollitropin alfa. Corifollitropin alfa has no LH activity, so the major difference between our protocol and current regimens using progestogen as a blockade of premature luteinization is whether the remaining LH activity under progestin suppression is adequate for ovarian stimulation in the first 7 days. Still widely debated is the necessity of LH supplementation for patients undergoing IVF/ICSI cycles. The uncertainty about the role of LH in ovarian stimulation continued due to conflicting results from different trials [27, 28]. The updated Cochrane meta-analysis showed no clear evidence of a difference between rLH combined with rFSH and rFSH alone in live birth rates despite more ongoing pregnancies under LH supplementation, in which the benefits appeared to be stronger for low responders [29]. Therefore, we applied the Elonva plus MPA regimen only in normal and high responders.

Earlier studies upon luteal phase stimulation using pure FSH components [3, 30, 31] usually started daily GnRH antagonist from the first day of follicle stimulation due to insufficient knowledge about the effect of endogenous progesterone on the prevention of LH surge. The combined suppressive effect of GnRH antagonists and endogenous progesterone in these regimens led to much more profound pituitary suppression and therefore might need higher dosage of FSH or longer stimulation duration, which is a totally different scenario from our protocol. Under PPOS without GnRH antagonist, the level of pituitary suppression is still to be elucidated. Hormone measurement in the PPOS study by Kuang et al. [9] demonstrated that the LH values gradually declined during ovarian stimulation, and the LH levels were suppressed after 5 days of MPA administration, which was compatible with prior contraception studies [32]. In our study, following 7 days of ovarian stimulation with a single shot of corifollitropin alfa and subsequent 6 days of MPA suppression, the LH levels were measured to be 2.70 ± 1.84 IU/L (data not shown), which was considered to be enough for folliculogenesis. After that, HMG was added until trigger criteria were met. Consequently, LH deficit may not be a critical issue in our protocol. Of course, further randomized controlled trials are needed to compare the pregnancy outcomes for our protocol and other published protocols.

To decrease the physical and mental stress from injection, various regimens were developed in the past. A single depot dose of long-acting GnRH agonist was expected to be superior to short-acting ones. Although no significant difference was concluded in the meta-analysis [33], the long-acting group was associated with not only higher OHSS risk but also inferior outcomes regarding superior-quality embryo rate and implantation rate in a retrospective study enrolling 478 patients [34]. Moreover, daily injection of gonadotropins are needed in the aforementioned down-regulation protocols. Wang et al. [35] proposed a patient-friendly corifollitropin alfa protocol using GnRH antagonist occasionally only when serum LH was ≧ 6 IU/L to reduce injections as much as possible. However, this method needs frequent blood withdrawals to monitor hormone levels for timely initiation of antagonist injection once LH rose above 6 IU/L, which is inconvenient and stressful on the other hand. Even under close follow-up, moreover, some LH peaks might have been missed. After all, according to the previous literature, there was a 20–25% of LH premature surge rate during ovarian stimulation without any preventative treatment [36]. In our regimen, the first visit was arranged at 7 days after corifollitropin alfa injection, and only an average of 1.4 visits were needed before trigger, which is convenient and friendly for the patients.

The unique advantage of our protocol is that seven daily injections are replaced by one shot of corifollitropin alfa as well as many injections of GnRH analogue can be omitted under oral MPA cotreatment, which is friendly for patients in many aspects including not only reduced mental/physical stress and time-consuming visits but also less cost (MPA vs. GnRH antagonist) and more convenience. We have also applied our corifollitropin alfa plus progestogen protocol in a random start fashion, which is needed on several occasions, e.g., emergent oocyte/embryo cryopreservation before chemotherapy and personal scheduling of patients themselves. Caution should be taken about the possibility of spontaneous pregnancy when ovarian stimulation is commenced in the late follicular and luteal phase. For most patients, we would recommend to initiate stimulation in early follicular phase.

Our study is limited by its study design that it is only an observational and proof-of-concept study. Further comparison studies, preferably randomized controlled trials, are required to clarify whether pregnancy outcomes of our friendly method are not inferior to those of conventional protocols.

## Conclusions

We have established a patient-friendly stimulation protocol for normal and high responders based on long-acting FSH and progestin, with an average of 3.6 shots and 1.4 visits before trigger and satisfactory reproductive outcomes.

## Abbreviations

AFC: antral follicle count
BMI: body mass index
CTP: carboxy-terminal peptide
DNA: Deoxyribonucleic acid
E2: estradiol
FET: frozen-thawed embryo transfer
FSH: follicular stimulation hormone
GnRH: Gonadotropin-releasing hormone
hCG: human chorionic gonadotropin
HMG: human menopausal gonadotropin
ICSI: intracytoplasmic sperm injection
IU: international unit
IVF: in vitro fertilization
Kg: kilogram
L: liter
LH: luteinizing hormone
MPA: Medroxyprogesterone acetate
OHSS: ovarian hyperstimulation syndrome
P: progesterone
PCOS: polycystic ovarian syndrome
PPOS: progestin-primed ovarian stimulation
rFSH: recombinant FSH

## References

[1] Maheshwari A, Gibreel A, Siristatidis CS, Bhattacharya S. Gonadotrophin-releasing hormone agonist protocols for pituitary suppression in assisted reproduction. Cochrane Database Syst Rev. 2011; 10.1002/14651858.CD006919.pub3. 10.1002/14651858.CD006919.pub3 21833958

[2] Al-Inany HG, Youssef MA, Ayeleke RO, Brown J, Lam WS, Broekmans FJ. Gonadotrophin-releasing hormone antagonists for assisted reproductive technology. Cochrane Database Syst Rev. 2016; 10.1002/14651858.CD001750.pub4. 10.1002/14651858.CD001750.pub4 PMC8626739 27126581

[3] von Wolff M, Thaler CJ, Frambach T, Zeeb C, Lawrenz B, Popovici RM, Strowitzki T (2009). Ovarian stimulation to cryopreserve fertilized oocytes in cancer patients can be started in the luteal phase. Fertil Steril.

[4] Wang N, Wang Y, Chen Q, Dong J, Tian H, Fu Y, Ai A, Lyu Q, Kuang Y (2016). Luteal-phase ovarian stimulation vs conventional ovarian stimulation in patients with normal ovarian reserve treated for IVF: a large retrospective cohort study. Clin Endocrinol.

[5] Boots CE, Meister M, Cooper AR, Hardi A, Jungheim ES (2016). Ovarian stimulation in the luteal phase: systematic review and meta-analysis. J Assist Reprod Genet.

[6] Kuang Y, Chen Q, Hong Q, Lyu Q, Fu Y, Ai A, Shoham Z (2013). Luteal-phase ovarian stimulation case report: three-year follow-up of a twin birth. J IVF Reprod Med Genet.

[7] Kuang Y, Hong Q, Chen Q, Lyu Q, Ai A, Fu Y, Shoham Z (2014). Luteal-phase ovarian stimulation is feasible for producing competent oocytes in women undergoing in vitro fertilization/intracytoplasmic sperm injection treatment, with optimal pregnancy outcomes in frozen-thawed embryo transfer cycles. Fertil Steril.

[8] Chen H, Wang Y, Lyu Q, Ai A, Fu Y, Tian H, Cai R, Hong Q, Chen Q, Shoham Z, Kuang Y (2015). Comparison of live-birth defects after luteal-phase ovarian stimulation vs. conventional ovarian stimulation for in vitro fertilization and vitrified embryo transfer cycles. Fertil Steril.

[9] Kuang Y, Chen Q, Fu Y, Wang Y, Hong Q, Lyu Q, Ai A, Shoham Z (2015). Medroxyprogesterone acetate is an effective oral alternative for preventing premature luteinizing hormone surges in women undergoing controlled ovarian hyperstimulation for in vitro fertilization. Fertil Steril.

[10] Zhu X, Zhang X, Fu Y (2015). Utrogestan as an effective oral alternative for preventing premature luteinizing hormone surges in women undergoing controlled ovarian hyperstimulation for in vitro fertilization. Medicine (Baltimore).

[11] Fares FA, Suganuma N, Nishimori K, LaPolt PS, Hsueh AJ, Boime I (1992). Design of a long-acting follitropin agonist by fusing the C-terminal sequence of the chorionic gonadotropin beta subunit to the follitropin beta subunit. Proc Natl Acad Sci U S A.

[12] Pouwer AW, Farquhar C, Kremer JA. Long-acting FSH versus daily FSH for women undergoing assisted reproduction. Cochrane Database Syst Rev. 2015; 10.1002/14651858.CD009577.pub3. 10.1002/14651858.CD009577.pub3 PMC10415736 26171903

[13] Massin N (2017). New stimulation regimens: endogenous and exogenous progesterone use to block the LH surge during ovarian stimulation for IVF. Hum Reprod Update.

[14] de Greef R, Zandvliet A, de Haan AF, Ijzerman-Boon PC, Marintcheva-Petrova M, Mannaerts BM (2010). Dose selection of corifollitropin alfa by modeling and simulation in controlled ovarian stimulation. Clin Pharmacol Ther.

[15] Veeck LL (1998). An atlas of human gametes and conceptuses.

[16] Soules MR, Steiner RA, Clifton DK, Cohen NL, Aksel S, Bremner WJ (1984). Progestin modulation of pulsatile luteinizing hormone secretion in normal women. J Clin Endocrinol Metab.

[17] Wildt L, Hutchison JS, Marshall G, Pohl CR, Knobil E (1981). On the site of action of progesterone in the blockade of the estradiol-induced gonadotropin discharge in the rhesus monkey. Endocrinology.

[18] Richter TA, Robinson JE, Lozano JM, Evans NP (2005). Progesterone can block the preovulatory gonadotropin-releasing hormone/luteinising hormone surge in the ewe by a direct inhibitory action on oestradiol-responsive cells within the hypothalamus. J Neuroendocrinol.

[19] Blockeel C, Drakopoulos P, Santos-Ribeiro S, Polyzos NP, Tournaye H (2016). A fresh look at the freeze-all protocol: a SWOT analysis. Hum Reprod.

[20] Messinis IE, Vanakara P, Zavos A, Verikouki C, Georgoulias P, Dafopoulos K (2010). Failure of the GnRH antagonist ganirelix to block the positive feedback effect of exogenous estrogen in normal women. Fertil Steril.

[21] Reichman DE, Zakarin L, Chao K, Meyer L, Davis OK, Rosenwarks Z (2014). Diminished ovarian reserve is the predominant risk factor for gonadotropin-releasing hormone antagonist failure resulting in breakthrough luteinizing hormone surges in in vitro fertilization cycles. Fertil Steril.

[22] Messinis IE, Loutradis D, Domali E, Kotsovassilis CP, Papastergiopoulou L, Kallitsaris A, Drakakis P, Dafopoulos K, Milingos S (2005). Alternate day and daily administration of GnRH antagonist may prevent premature luteinization to a similar extent during FSH treatment. Hum Reprod.

[23] Wang Y, Chen Q, Wang N, Chen H, Lyu Q, Kuang Y (2016). Controlled ovarian stimulation using Medroxyprogesterone acetate and hMG in patients with polycystic ovary syndrome treated for IVF: a double-blind randomized crossover clinical trial. Medicine (Baltimore).

[24] Dong J, Wang Y, Chai WR, Hong QQ, Wang NL, Sun LH, Long H, Wang L, Tian H, Lyu QF, Lu XF, Chen QJ, Kuang YP (2017). The pregnancy outcome of progestin-primed ovarian stimulation using 4 versus 10 mg of medroxyprogesterone acetate per day in infertile women undergoing in vitro fertilisation: a randomised controlled trial. BJOG.

[25] Chen Q, Wang Y, Sun L, Zhang S, Chai W, Hong Q, Long H, Wang L, Lyu Q, Kuang Y (2017). Controlled ovulation of the dominant follicle using progestin in minimal stimulation in poor responders. Reprod Biol Endocrinol.

[26] Fauser BC, Mannaerts BM, Devroey P, Leader A, Boime I, Baird DT (2009). Advances in recombinant DNA technology: corifollitropin alfa, a hybrid molecule with sustained follicle-stimulating activity and reduced injection frequency. Hum Reprod Update.

[27] Hill MJ, Levens ED, Levy G, Ryan ME, Csokmay JM, DeCherney AH, Whitcomb BW (2012). The use of recombinant luteinizing hormone in patients undergoing assisted reproductive techniques with advanced reproductive age: a systematic review and meta-analysis. Fertil Steril.

[28] Xiong Y, Bu Z, Dai W, Zhang M, Bao X, Sun Y (2014). Recombinant luteinizing hormone supplementation in women undergoing in vitro fertilization/ intracytoplasmic sperm injection with gonadotropin releasing hormone antagonist protocol: a systematic review and meta-analysis. Reprod Biol Endocrinol.

[29] Mochtar MH, Danhof NA, Ayeleke RO, Van der Veen F, van Wely M. Recombinant luteinizing hormone (rLH) and recombinant follicle stimulating hormone (rFSH) for ovarian stimulation in IVF/ICSI cycles. Cochrane Database Syst Rev. 2017; 10.1002/14651858.CD005070.pub3. 10.1002/14651858.CD005070.pub3 PMC6481753 28537052

[30] Buendgen NK, Schultze-Mosgau A, Cordes T, Diedrich K, Griesinger G (2013). Initiation of ovarian stimulation independent of the menstrual cycle: a case-control study. Arch Gynecol Obstet.

[31] Martínez F, Clua E, Devesa M, Rodríguez I, Arroyo G, González C, Solé M, Tur R, Coroleu B, Barri PN (2014). Comparison of starting ovarian stimulation on day 2 versus day 15 of the menstrual cycle in the same oocyte donor and pregnancy rates among the corresponding recipients of vitrified oocytes. Fertil Steril.

[32] Wikström A, Green B, Johansson ED (1984). The plasma concentration of medroxyprogesterone acetate and ovarian function during treatment with medroxyprogesterone acetate in 5 and 10 mg doses. Acta Obstet Gynecol Scand.

[33] Albuquerque LET, Tso LO, Saconato H, Albuquerque MCRM, Macedo CR. Depot versus daily administration of gonadotrophin-releasing hormone agonist protocols for pituitary down regulation in assisted reproduction cycles. Cochrane Database Syst Rev. 2013; 10.1002/14651858.CD002808.pub3. 10.1002/14651858.CD002808.pub3 PMC7133778 23440788

[34] Duan L, Bao S, Li K, Teng X, Hong L, Zhao X (2017). Comparing the long-acting and short-acting forms of gonadotropin-releasing hormone agonists in the long protocol of IVF/ICSI cycles: a retrospective study. J Obstet Gynaecol Res.

[35] Wang HL, Lai HH, Chuang TH, Shih YW, Huang SC, Lee MJ, Chen SU (2016). A patient friendly Corifollitropin alfa protocol without routine pituitary suppression in normal responders. PLoS One.

[36] Eibschitz I, Belaish-Allart J, Frydman R (1986). In vitro fertilization management and the results in stimulated cycles with spontaneous luteinizing hormone discharge. Fertil Steril.

